# Supplementary material for: Incidence and Impact of Acute Kidney Injury in Patients Receiving Extracorporeal Membrane Oxygenation: A Meta-Analysis
Source: J Clin Med. 2019 Jul 5;8(7):981. doi: 10.3390/jcm8070981 (PMC6678289; doi:10.3390/jcm8070981)
Supplement: Supplementary file 1 [file jcm-08-00981-s001.pdf]

Online supplementary data 1

Search terms for systematic review.

Database: Ovid MEDLINE (747 articles)

1. exp acute kidney injury/
2. acute kidney injury$.mp
3. exp acute renal failure/
4. acute renal failure$.mp.
5. exp renal insufficiency/
6. renal insufficiency$.mp.
7. exp dialysis/
8. dialysis$.mp.
9. hemodialysis$.mp.
10. renal replacement therapy$.mp.
11. hemofiltration$.mp.
12. hemodiafiltration$.mp.
13. 1 or 2 or 3 or 4 or 5 or 6 or 7 or 8 or 9 or 10 or 11 or 12
14. Extracorporeal Membrane Oxygenation.mp
15. Exp Extracorporeal Membrane Oxygenation/
16. ECMO.mp
17. 14 or 15 or 16
18. 13 and 17

Database: EMBASE (879 articles)

('extracorporeal oxygenation' OR 'extracorporeal membrane oxygenation device' OR 'extracorporeal membrane oxygenation cannula') AND 'acute kidney failure'

Cochrane Database (6 articles)

"extracorporeal membrane oxygenation" AND "acute kidney injury"
